# The Impact of Depression Symptoms in Patients with Parkinson’s Disease: A Novel Case-Control Investigation

**DOI:** 10.3390/ijerph18052369

**Published:** 2021-03-01

**Authors:** Ana María Jiménez-Cebrián, Ricardo Becerro-de-Bengoa-Vallejo, Marta Elena Losa-Iglesias, Daniel López-López, César Calvo-Lobo, Patricia Palomo-López, Carlos Romero-Morales, Emmanuel Navarro-Flores

**Affiliations:** 1 Department Nursing and Podiatry, Faculty of Health Sciences, Instituto de Investigación Biomédica de Málaga (IBIMA), Ampliación de Campus de Teatinos, University of Malaga, Arquitecto Francisco Peñalosa 3, 29071 Málaga, Spain; amjimenezc@uma.es; 2 Facultad de Enfermería, Fisioterapia y Podología, Universidad Complutense de Madrid, 28040 Madrid, Spain; ribebeva@ucm.es (R.B.-d.-B.-V.); cescalvo@ucm.es (C.C.-L.); 3 Faculty of Health Sciences, Universidad Rey Juan Carlos, 28933 Alcorcón, Spain; marta.losa@urjc.es; 4 Research, Health and Podiatry Group, Department of Health Sciences, Faculty of Nursing and Podiatry, Universidade da Coruña, 15403 Ferrol, Spain; daniellopez@udc.es; 5 University Center of Plasencia, Universidad de Extremadura, 10600 Plasencia, Spain; patibiom@unex.es; 6 Faculty of Sport Sciences, Universidad Europea de Madrid, Villaviciosa de Odón, 28670 Madrid, Spain; 7 Frailty Research Organized Group, Department of Nursing, Faculty of Nursing and Podiatry, Universidad de Valencia, 46010 Valencia, Spain; manu.navarroflores@gmail.com

**Keywords:** beck depression inventory, depression, Parkinson disease

## Abstract

Parkinson’s disease is a common neurodegenerative disease and it is known to cause motor disturbances associated with musculoskeletal problems of the locomotor apparatus, and non-motor symptoms, that are believed to have a harmful effect on health, social functioning and mobility. The aim of this study was to evaluate depression in patients with Parkinson’s Disease (PD) compared to subjects who do not have it. The sample consisted of 124 participants (mean age 69.18 ± 9.12). Patients with PD were recruited from a center of excellence for Parkinson’s disease (cases *n* = 62) and healthy subjects without PD from their relatives and caregivers (control *n* = 62). The Spanish version of Beck’s Depression Inventory (BDI) scores and categories were collected. A clear statistically significant difference (*p* < 0.05) was evident in the BDI scores between both groups. Parkinson’s patients presented worse results on the BDI = 15.48 ± 7.24 points compared to healthy subjects with BDI = 7.03 ± 6.99 points. Regarding BDI categories, there were statistically significant differences (*p* < 0.001) for the greater BDI categories in the Parkinson’s group compared with healthy subjects. The depression represents an important potential risk for increased symptoms and negative impact among patients with PD compared with healthy subjects.

## 1. Introduction

Parkinson’s disease (PD) is the second most common neurodegenerative disease in older people after Alzheimer’s disease. A prevalence of 0.3% is estimated in the general population, reaching 2% in those older than 60 years and higher than 4% in those older than 80 years. The estimated incidence is between 8 and 18 cases per 100,000 inhabitants/year. Spain has an incidence and prevalence similar to the rest of Europe [1,2].

Although PD is defined primarily by motor disturbances such as slowed or involuntary movements such as tremors, non-motor symptoms such as anxiety, depression, fatigue, apathy, sleep disturbances, and sensory symptoms may also accompany the disease [3].

In PD, depression is a non-motor symptom that can precede the onset of motor symptoms [4]. The presence of depression in the early stages of PD and prior to motor symptoms appears to be related to changes in the structure of the brain, such as the pathological involvement of monoaminergic nuclei in brainstem cerebrovascular disease, Alzheimer disease, Lewy body pathology, functional changes in the limbic and subcortical circuits, hippocampal atrophy, alterations of neurogenesis and neurotrophic factors, and toxic stress, with hypercortisolemia and inflammation [5].

There are studies that confirm the hypothesis that depression is part of the disease, being a prodromal symptom in PD and not as a risk factor for the disease [6,7]. The etiology of depression in PD is unclear, and the changes in brain neurotransmitters in combination with the subjective reaction to it may contribute [8].

Depression in patients with PD is related to motor symptoms. De novo PD shows severe levels of depression associated with a greater motor deficit and lesser cognitive deficit. However, during the follow-up of the disease over time, the presence of severe depression is associated with the administration of high doses of levodopa in the control of motor symptoms [4].

The diagnosis of depression in PD is not always easy due to the overlap of psychopathological symptoms such as apathy and clinical manifestations such as bradykinesia and hypomimia. The general prevalence of depression in Parkinson’s patients is between 30 and 35% [5]. Although there are differences according to authors, Almeida et al., consider prevalence of depression to be between 50 and 70% in patients with PD [9]. A study with a large sample showed that prevalence was higher in women than in men [10,11]. Lower prevalence figures are obtained when the different clinical contexts and different diagnostic approaches are taken into account; such is the case of the results obtained from a systematic review on the prevalence of depression in Parkinson’s patients, which determines mild depression in 22% (the most frequent) and major depression in 17% [12].

Psychosocial factors and pain could also play a role in depression. Parkinson’s patients suffer from chronic musculoskeletal or neuropathic pain. Pain is a strong predictor of depression and vice versa. A close association between pain and depression has been identified in PD [5]. Successful pain management is essential to successfully treating depression [13].

Despite this, no studies have been carried out so far to analyze depression in PD and non-PD individuals and to prevent postural instability, gait difficulties, falls and other basic illnesses in the attempt to find a better quality of life and wellbeing for patients with PD.

This study aims to evaluate depression in patients with PD compared to subjects who do not have PD.

## 2. Material and Methods

### 2.1. Design and Sample

A total sample of 124 subjects was recruited for this investigation. A descriptive and observational case–control study was carried out in a center of excellence for PD in Malaga (Spain) between October 2020 and December 2020. Study subjects consisted of Parkinson’s patients (case group *n* = 62) and non-Parkinson’s (control group *n* = 62). Patients were recruited by a consecutive sampling method using a successive and non-randomized sample method. Subjects who suffered PD were enlisted from the PD center of excellence and control subjects were recruited from their Clinic of Podiatric Medicine and Surgery. The selection for participation and inclusion were: (1) age over 50, (2) persons with PD (case group) and persons without PD (control group). The exclusion criteria were: (1) people with a diagnosis of depression, (2) refusal to supply informed consent and (3) inability to understand and carry out the instructions in the research.

### 2.2. Procedure

Baseline measurements included general questions associated with (1) demographic variables (age, weight, height, job status, level of studies and marital status); and (2) characteristics of comorbid conditions (diabetes, obesity, musculoskeletal difficulties, and vascular disorders). In addition, exact health questions associated with PD such as (3) stage of the PD and (4) years of suffering the disease, were recorded. Next, participants completed the Spanish Beck Depression Inventory (BDI) questionnaire [13,14,15]. This instrument was translated into Spanish and has been evidenced to be a validated instrument used for the assessment of depression. It comprises 21 questions and each item is scored on a sub-scale between zero and three points, giving a global result between 0 and 63 points. The analyses of the results were recorded according to the following categories: (1) from 0 to 13 as no sign of depression, (2) 14 to 19 as mild depression, (3) 20 to 28 as moderate depression, (4) 24 to 63 as severe depression. This instrument can be considered as an easy and accurate test for the evaluation of subjects with signs of depression. The internal consistency of the BDI demonstrates a Cronbach’s alpha coefficient of 0.889 [16,17].

### 2.3. Ethical Considerations

The current study received a positive report issued by the Bioethics and Biosafety Committee at the University of Valencia (Valencia, Spain, 2020; Code: 1450610). All volunteers gave written informed consent before being part of this investigation. The ethical standards for human experimentation of the Declaration of Helsinki (World Medical Association) and other organizations were respected at all times [18].

### 2.4. Sample Size Calculation

The sample size was calculated for this study of cases and controls with specific levels of confidence, power, and groups of equal size using the Epidat 4.2 (Program. Consellería de Sanidade, Xunta de Galicia, Spain; Organización Panamericana de la salud (OPS-OMS); Universidad CES, Colombia). A total sample size of 122 participants (61 per group) was established, taking a confidence level of 70%, a power of 0.80, an odds ratio of 2.0 and an expected proportion of exposed of 66, 67%, and 50% in the controls. The total sample (124 participants) consisted of 62 cases (38 men and 24 women) and 62 controls (37 men and 25 women).

### 2.5. Statistical Analysis

The statistical analysis was performed using 25.0 v SPSS software (IBM Corp., Armonk, NY, USA) referring to an alpha error of 0.05 for a 95% confidence interval (CI).

Regarding quantitative data, the Kolmogorov-Smirnov test was used to evaluate normality. All data were shown as parametric data (Kolmogorov–Smirnov test showed a *p*-value lower than 0.05) and were described as mean ± standard deviation (SD) and range (minimum–maximum), and contrasts between both groups were compared with the Student’s and Mann–Whitney U tests for independent samples.

Concerning categorical data, frequencies and percentages were applied to distinguish these values, and differences between both groups were contrasted with the Chi squared test (BDI category).

## 3. Results

### 3.1. Descriptive Data

A sample of 124 subjects completed the research and was divided into persons with PD (for case group, *n* = 62) and healthy matched-paired participants (for the control group, *n* = 62) showing an age division from 50 to 84 years old. Statistically significant differences were not shown (*p* > 0.05) between both groups for descriptive data (Table 1).

### 3.2. Outcome Measurements

Generally speaking, 100% (*n* = 124) of participants presented the characteristics that are shown in Table 1. A subsequent physical examination revealed in the cases group 56.4% (*n* = 35) had joint stiffness, 12.9% (*n* = 8) had keratosis, 41.9% (*n* = 26) had foot pain, and 27.4% (*n* = 17) had deformed toes; and in the control group 1.6% (*n* = 1) had joint stiffness, 1.6% (*n* = 1) had keratosis, 16.1% (*n* = 10) had foot pain, and 3.2% (*n* = 2) had deformed toes. Furthermore, 54.8% of the patients who participated in the research presented predisposing factors such as 21.8% (*n* = 27) vascular disease, 21.8% (*n* = 27) osteoarticular pathology, 9.7% (*n* = 12) diabetes and 4% (*n* = 5) obesity.

A clear statistically significant difference (*p* < 0.05) was present for the BDI scores between both groups (Table 2). Regarding the results, subjects who suffered from PD presented worse results on the BDI = 15.48 ± 7.24 points (higher BDI scores) compared to healthy subjects with BDI = 7.03 ± 6.99 points (lower BDI results). Regarding the BDI categories, there were statistically significant differences (*p* < 0.001) for greater BDI categories in the PD group compared with healthy subjects (Table 2).

## 4. Discussion

This is a first case control study in Spain that compares the depression scores obtained from 62 subjects with PD and 62 without PD (considered healthy control participants). The results found show that the majority of the subjects with PD in this research suffer from depression (58%) at one of its three levels with respect to control group.

Therefore, PD individuals should be evaluated and monitored to initially detect depressive symptoms. Parkinson’s disease requires a multidisciplinary approach for promoting both physical and psycho-logical well-being [19].

This prevalence is close to that of another descriptive study on depression in people over 50 years of age, where 57.4% of PD patients suffered from depression [20]. Likewise, the systematic review carried out by Almeida et al. on depression and chronic diseases contains a section in which it analyzes depression in patients with PD. In its analyses it estimates that the prevalence of depression in these patients varies according to the studies, although it is estimated that between 50 and 70% of patients with PD are affected by depression [9].

Similarly, our results coincide with those of other studies of depression in patients with PD. In the research by Zhang et al., they studied the non-motor symptoms of this disease, analyzing depression in a large sample of subjects with PD, resulting in an incidence of depression of around 41.5%, while mental and behavior disorders obtained a low incidence [21]. It is confirmed that anxiety and depression in any of its stages are more frequent in patients with neurological conditions than with other somatic conditions [22].

Ehrt et al. compared two groups of patients with depression, one suffered from PD and the other was without PD. It was shown that the type of depression in the PD group differed from the group that did not suffer from it, with less feelings of sadness and guilt in the PD group but with more mental concentration problems. It seems that this could be related to the brain changes in PD, which influence the clinical picture of depression [23]. Brain variations are structural and functional, with loss of white matter within the cortical-limbic network positively associated with depression [5].

In our study, two groups were also compared, subjects with PD and without PD, but with a different approach, not all participants had depression as an inclusion criterion. Results showed that in subjects with PD, the type of depression found most frequently was classified as mild (30.6%).

In our research, the level of depression in the participants was measured with the Beck Depression Inventory (BDI) questionnaire. This questionnaire has been recognized by other authors as having optimal specificity and sensitivity for measuring depression in PD patients [24]. However, Zhang et al. and Ehrt et al., used different questionnaires for the measurement and classification of depression (Hamilton Depression Scale and Montgomery-Asberg Depression Rating Scale, respectively) [21,23].

The variations found among different studies of depression carried out on PD patients may, among other factors, be due to the use of different methods for assessing depression and the type of sample chosen.

However, in the study carried out by López-López et al., the Beck Depression Inventory Questionnaire was used to compare the levels of depression in patients with asthma with respect to healthy subjects, not finding significant differences between both groups. Similarities can be highlighted with our study, in the use of the same questionnaire, and the group of patients studied, with asthma, which is a chronic disease like PD [25]. In 2004, the World Health Organization included comorbidity between depression and chronic physical illness among its ten global public health concerns. A bidirectional association between the two was established, with greater relevance in old age [26]. In our study, these two aspects converge.

Few studies have explored the timing of onset of depression relative to that of PD, and it is essential to determine if depression is a prodromal condition of PD (in which case the time lag between onset of depression and onset of PD would be short) or whether it is a risk factor for PD (in which case an extended time lag between onset of depression and onset of PD would be expected) [5].

Finally, considering these findings, it is important that PD patients know the link with depression, how to find out in the initial stage and are instructed in its recognition and prevention. The actions of different health professionals in a multidisciplinary care team with shared clinical decisions may improve the management of this disease and the quality of life of patients [27].

This investigation had some limitations. Although the BDI may be regarded as a valid and reliable test and was applied cross-culturally in Europe, particular caution should be considered in the population of Spain. In fact, regression analyses determined the inconsistency of the population of Spain with respect to other population of the other European populations related with the following items of the tool 3, 6, 7, 9, 13, 15, and 21 [28]. Thus, the use of two or more tools in conjunction with BDI could consolidate the quality of new studies and can help to recognize the presence of a culture where this link does not appear to be recognized as an involved mechanism.

## 5. Conclusions

The depression represents an important potential risk for increased symptoms and negative impact among patients with PD compared with healthy subjects.

## Figures and Tables

**Table 1:** Descriptive data of the Parkinson’s patients and healthy matched-paired controls.

Descriptive Data		Total Group Mean ± SD Range (*n* = 124)	Cases Mean ± SD Range (*n* = 62)	Controls Mean ± SD Range (*n* = 62)	*p*-Value
Age (years)		69.18 ± 9.12 (50–84)	69.23 ± 9.15 (50–84)	69.13 ± 9.15 (50–84)	0.097 ^†^
Weight (kg)		74.10 ± 14.84 (43–135)	73.36 ± 17.63 (43–135)	74.83 ± 11.49 (54–100)	0.582 ^†^
Height (m)		1.67 ± 0.09 (1.47–1.91)	1.66.37 ± 9.64 (1.47–1.91)	1.67 ± 7.80 (1.47–1.85)	0.690 ^†^
BMI (kg/m^2^)		26.61 ± 4.61 (16.16–40.31)	26.37 ± 5.24 (16.16–40.31)	26.85 ± 3.90 (19.83–35.43)	0.0563 ^†^
Sex (%)	Male Female	75 (60.5%) 49 (39.5)	38 (61.3%) 24 (38.7%)	37 (59.7%) 25 (40.3)	0.854 ^‡^

Abbreviations: BMI, body mass index; SD, standard deviation. In all the analyses, *p* < 0.05 (with a 95% confidence interval) was considered statistically significant. median ± interquartile range, range (min–max) and ^†^ Student’s *t*-test for independent samples were applied. ^‡^ Chi-squared test were used.

**Table 2:** Comparisons of BDI scores and categories between the Parkinson’s patients and healthy matched-paired controls.

Outcome Measurements		Total Group (*n* = 124)	Cases Mean ± SD (*n* = 62)	Controls (*n* = 62)	*p*-Value (Cases vs. Controls)
BDI Category *	No Depression	76 (61.3%)	26 (41.9%)	50 (80.6%)	0.001 *
	Mild	27 (21.8%)	19 (30.6%)	8 (12.9%)	
	Moderate	16 (12.9%)	14 (22.6%)	2 (3.2%)	
	Severe	5 (4%)	3 (4.8%)	2 (3.2%)	
BDI scores		11.26 ± 8.26 (0.00–32.00)	15.48 ± 7.24 (3.00–32.00)	7.03 ± 6.99 (0.00–30.00)	**<0.001 ^†^**

* BDI, Beck depression inventory. Frequency, percentage (%) and Chi-squared test (χ^2^) were utilized. BDI domains were divided as: (1) 0 to 9 points without depression, (2) 10 to 15 points: mild depression, (3) 16 to 23 points: moderate depression, (4) 24 to 57 points: severe depression. **^†^** BDI scores, Median ± interquartile range, range (min–max) and Mann–Whitney U test were used. In all the analyses, *p* < 0.05 (with a 95% confidence interval) was considered statistically significant (bold).
